# Material type versus defect size in adult cranioplasty: a multi-institutional analysis of 30-day outcomes and risk prediction modeling^☆^

**DOI:** 10.1016/j.jpra.2026.08.024

**Published:** 2026-08-19

**Authors:** Jennifer A. Watson, Julius M. Wirtz, Victoria Kong, Samuel A. Knoedler, Leonard Knoedler, Robert Gaudin, Herrmann F. Sailer, Elisa Colombo, Schirin Rahiminia, Bahar Bassiri Gharb, Giuseppe Esposito, Antonio Rampazzo, Filippo A.G. Perozzo

**Affiliations:** a Department of Plastic Surgery and Hand Surgery, University Hospital Zurich, Zurich, Switzerland; b UCSF Medical Center, University of California San Francisco, San Francisco, CA, USA; c Division of Plastic Surgery, Department of Surgery, Yale School of Medicine, New Haven, CT, USA; d Department of Plastic Surgery and Hand Surgery, Klinikum Rechts der Isar, Technical University of Munich, Munich, Germany; e Charité – Universitätsmedizin Berlin, Corporate Member of Freie Universität Berlin, Humboldt-Universität zu Berlin, and Berlin Institute of Health, Department of Oral and Maxillofacial Surgery, Berlin, Germany; f Department of Oral and Maxillofacial Surgery, SailerClinic, Zurich, Switzerland; g Private University in the Principality of Liechtenstein, Institute of Postgraduate Dentistry, Liechtenstein; h Department of Neurosurgery, University Hospital Zurich, Zurich, Switzerland; i Department of Plastic and Reconstructive Surgery, Cleveland Clinic Foundation, Cleveland, OH, USA; j Division of Plastic Surgery, Department of Surgery, University of Pennsylvania, Philadelphia, PA, USA

**Keywords:** Cranioplasty, Alloplastic materials, Autologous bone, Defect size, Postoperative complications, Risk prediction

## Abstract

**Background:**

The optimal cranioplasty material remains controversial in the context of defect size and patient risk. We analyzed multi-institutional data over 13 years to evaluate the interaction between material type and defect size.

**Methods:**

We performed a retrospective cohort analysis of adult cranioplasty patients in the ACS-NSQIP (2011–2023), stratifying by material type (autograft vs. alloplastic) and defect size (≤5 cm vs. >5 cm). The primary outcome was any 30-day complication. Multivariable logistic regression adjusting for 21 covariates, interaction analyses, and machine learning models were applied.

**Results:**

Among 3894 patients, 88% underwent autograft and 12% alloplastic cranioplasty. The 30-day complication rate was 22%, with no significant difference between material types (*p* = 0.72). On multivariable analysis, material type was not independently associated with complications (*p* = 0.715); defect size >5 cm was an independent predictor (aOR 1.24, *p* = 0.032). For large defects, alloplastic reconstruction reduced operative time by 90 min (189 vs. 279 min; *p* < 0.001). Nine independent predictors were identified; ventilator dependence (aOR 8.44) and preoperative transfusion (aOR 4.35) were strongest. Alloplastic use increased five-fold while adjusted complication risk decreased by 4.5% annually. Logistic regression achieved the highest predictive performance (AUC 0.737).

**Conclusion:**

Material type is not an independent predictor of short-term complications following cranioplasty, regardless of defect size or patient risk profile. Larger defects are associated with increased morbidity, but alloplastic materials offer significant operative efficiency without compromising short-term perioperative safety. Prospective studies with extended follow-up stratified by alloplastic subtype are needed.

## Background

Cranioplasty is one of the oldest documented surgeries and serves an integral role in modern neurosurgical practice.^1^^,^ ^2^ Beyond its reconstructive aims, cranioplasty serves critical functional roles, including the restoration of cerebrospinal fluid dynamics, normalization of intracranial pressure, and the protection of underlying neural structures.^3^^,^^4^ Furthermore, cranioplasty contributes significantly to neurological recovery and psychosocial well-being by restoring cranial contour and symmetry.^5^^,^^6^ However, despite its routine use and history, this procedure is associated with a disproportionately high complication rate up to 50%.^1^ Therefore, there is a need for evidence-based guidelines regarding the optimal approach to subsequent reconstruction.

The choice of reconstructive material for cranioplasty remains a point of contentious debate. Traditionally, autologous bone has been regarded as the gold standard owing to its inherent biocompatibility, low immunogenicity, low complication rates, and high success rates.^1^ However, its use is limited by donor-site morbidity, risk of contamination during storage, unpredictable resorption, and restricted availability in the setting of comminuted fractures or infection.^2^^,^^7^^,^^8^ These limitations are particularly relevant in complex or large cranial defects, where sufficient viable bone may not be available.

With the rapid evolution in alloplastic technology, including the use of polymethylmethacrylate (PMMA), polyetheretherketone (PEEK), porous polyethylene and titanium, this designation is increasingly contested.^8^^,^^9^ These materials offer several advantages, such as the ability to pre-fabricate patient-specific implants using computer-aided design and three-dimensional (3D) printing technologies.^1^^,^^10^, ^11^, ^12^ This allows for precise reconstruction of large and complex defects, reducing intraoperative contouring and therefore overall operative time. Yet, concerns regarding an increased risk of surgical site infection and implant extrusion with synthetic materials persist in the literature.^1^^,^^9^^,^^10^ As a result, the relative safety and efficacy of autologous versus alloplastic reconstruction remain controversial.

Current evidence comparing autograft to alloplastic materials is largely inconclusive, often limited by small, single-center cohorts, and a failure to account for the confounding influence of defect size.^2^^,^^6^^,^^12^ Furthermore, while the technical complexity of the procedure is known to scale with the magnitude of the calvarial void, the interaction between material type and defect size on short-term outcomes has not been systematically evaluated.^13^ Larger defects are more likely to be reconstructed with alloplastic materials due to the limited availability or insufficiency of autologous bone, introducing inherent selection bias.^14^ As the global use of alloplastic materials continues to expand, there is a critical need to determine whether these synthetic substitutes provide comparable safety profiles to autologous bone across varying patient risk strata.^8^ Addressing this question is essential for refining surgical algorithms and ensuring optimal patient outcomes.

To address these gaps, the present study leverages the American College of Surgeons National Surgical Quality Improvement Program (ACS-NSQIP), a large, multi-institutional clinical database, to perform a comprehensive analysis of cranioplasty outcomes. Specifically, we aimed to (i) evaluate the independent effects of reconstructive material and defect size on 30-day morbidity and mortality; (ii) examine potential interactions between these variables across distinct clinical risk profiles; (iii) characterize temporal trends in material utilization over the study period; and (iv) develop and internally validate a clinically applicable predictive model for postoperative complications.

## Methods

### Data source

A retrospective cohort analysis was conducted using the ACS-NSQIP Participant Use Data Files (PUF) from 2011 through 2023. ACS-NSQIP is a validated, risk-adjusted, multi-institutional registry that collects standardized perioperative data from hundreds of participating hospitals. Participating institutions include academic medical centers and community hospitals across the United States enrolled in the ACS-NSQIP program. The database is well-established in outcomes research and includes >150 variables encompassing patient demographics, comorbidities, intraoperative characteristics, and 30-day postoperative outcomes, all defined according to strict, uniform criteria and abstracted by trained clinical reviewers.^15^, ^16^, ^17^, ^18^, ^19^ The high data fidelity, standardized definitions, and rigorous auditing processes of ACS-NSQIP ensure reproducibility and reliability. All data were de-identified prior to analysis; therefore, this study was exempt from Institutional Review Board approval. This study was reported in accordance with the Strengthening the Reporting of Observational Studies in Epidemiology (STROBE) guidelines for cohort studies. A completed STROBE checklist is provided as a supplementary appendix.

### Patient selection

**Fig. 1:**
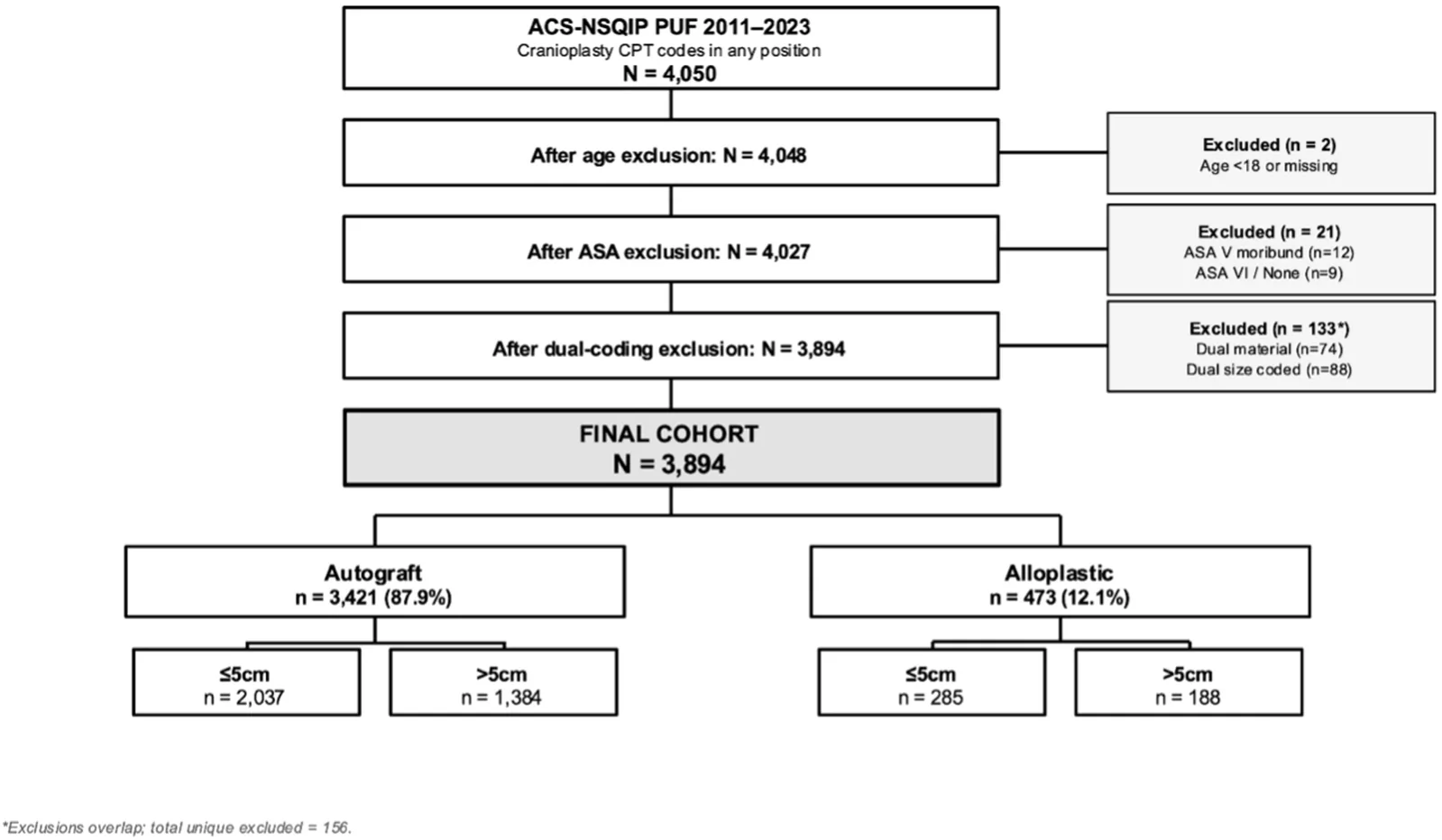
Patient Flow Diagram. Study cohort selection from the American College of Surgeons National Surgical Quality Improvement Program (ACS-NSQIP) Participant Use Files, 2011–2023. Cranioplasty cases were identified using Current Procedural Terminology (CPT) codes 62,140, 62,141, 62,146, and 62,147. After exclusion of 156 patients (age <18 or missing, ASA class V or unassigned, dual material or dual size coding), the final cohort comprised 3894 patients stratified by material type (autograft vs. alloplastic) and defect size (≤5 cm vs. >5 cm). *Exclusions overlap; total unique excluded = 156.

Adult patients (≥18 years) undergoing cranioplasty were identified using Current Procedural Terminology (CPT) codes 62,140, 62,141, 62,146, and 62,147. Cases were excluded if they were pediatric or if they had dual material or dual defect size coding. Cases with American Society of Anesthesiologists (ASA) physical status classification V were also excluded. To ensure complete ascertainment, cases with missing key variables, including age and ASA class, were excluded. See Fig. 1.

### Variable extraction

The primary exposure was categorized by material type (autograft vs. alloplastic) and defect size (≤5 cm vs. >5 cm). Patients and procedures were stratified into four categories based on reconstructive material (alloplastic [CPT 62,140, 62,141] or autologous [CPT 62,146, 62,147]) and defect size (≤5 cm [CPT 62,140, 62,146] or >5 cm [CPT 62,141, 62,147]). The primary outcome was a composite 30-day ‘any complication’ variable, defined as any surgical complication (surgical site infection (SSI) or dehiscence), medical complication (pneumonia, unplanned reintubation, pulmonary embolism, ventilator use >48 h, deep vein thrombosis (DVT), urinary tract infection (UTI), myocardial infarction (MI), cardiac arrest, stroke, sepsis, or septic shock), or bleeding requiring transfusion, return to the operating room, or 30-day mortality. Secondary outcomes included each component of the composite outcome, 30-day readmission, and non-home discharge. Twenty-one covariates were extracted, including patient demographics, comorbidities, functional status, ASA class, and operative characteristics.

### Statistical analysis

All analyses were conducted in R version 4.5.1 (R Foundation for Statistical Computing, Vienna, Austria) using the tidyverse, gtsummary, pROC, glmnet, randomForest, xgboost, and rms packages. Statistical significance was defined as a two-sided *p* < 0.05. Continuous variables are reported as mean ± standard deviation and compared using the independent samples *t*-test for two-group comparisons and one-way analysis of variance (ANOVA) for four-group comparisons. Categorical variables are presented as frequencies with percentages and compared using chi-square tests or Fisher's exact test, as appropriate. Univariable logistic regression was performed to screen for associations between preoperative and procedural variables and the primary outcome of any 30-day complication. All clinically relevant variables were subsequently entered into a multivariable logistic regression model adjusting for 21 covariates: age, sex, body mass index (BMI), diabetes mellitus, current smoking status, ventilator dependence, chronic obstructive pulmonary disease (COPD), congestive heart failure (CHF), hypertension requiring medication, dialysis, disseminated cancer, chronic steroid use, bleeding disorder, preoperative transfusion, functional dependence, American Society of Anesthesiologists (ASA) physical status classification, operative time, emergency status, inpatient setting, material type, and defect size. With 852 outcome events available for 21 covariates, the events-per-variable ratio (approximately 40) substantially exceeded the conventional minimum of 10, indicating that the model was not over-parameterized. Model discrimination was assessed using the area under the receiver operating characteristic curve (C-statistic). The final multivariable model achieved a C-statistic of 0.74 with bootstrap-validated calibration; because the model was intended for individualized risk estimation rather than binary classification, no operating-point threshold on the ROC curve was optimized. Results are reported as adjusted odds ratios (aORs) with 95% confidence intervals (CIs). A complete-case analysis was performed; 2821 of 3894 patients (72%) had complete data across all covariates. All 21 covariates were selected a priori based on clinical relevance and prior literature, and entered simultaneously into the multivariable model regardless of univariable significance. To evaluate potential effect modification, multiplicative interaction terms were tested between material type and defect size (material × defect size) and between material type and ASA risk category (material × ASA class). Stratified multivariable analyses were additionally performed within each defect size group (≤5 cm and >5 cm) and within ASA risk strata (Low: I–II vs. High: III–IV) to quantify the adjusted effect of alloplastic material within each subgroup. Temporal trends in material utilization, complication rates, and operative duration across the 13-year study period were assessed using Cochran-Armitage tests for trend. The independent effect of calendar year on complication risk was evaluated by including year as a continuous covariate in the fully adjusted multivariable model. Predictive performance for the primary outcome was benchmarked across four machine learning architectures: logistic regression, least absolute shrinkage and selection operator (LASSO), extreme gradient boosting (XGBoost), and random forest. These machine-learning models were included as a secondary benchmarking exercise to test whether more flexible algorithms could improve discrimination beyond conventional regression; the logistic regression model remained the primary, pre-specified analysis, and the machine-learning comparison is intended to support rather than supplant the clinical message. Data were split into training (80%) and test (20%) sets with a fixed random seed for reproducibility. Discriminative performance was evaluated using AUC on the held-out test set, with 95% confidence intervals derived from DeLong's method. Model calibration was assessed for the logistic regression model using 200 bootstrap resamples, and a clinical nomogram was developed from this model to enable individualized risk prediction. No formal sample size calculation was performed; the full eligible cohort of 3894 patients identified from ACS-NSQIP 2011–2023 was included. As ACS-NSQIP captures structured 30-day outcomes for all enrolled cases through trained clinical reviewers, loss to follow-up is not applicable to this study. Given the large number of secondary outcomes and subgroup comparisons examined, no formal correction for multiple comparisons was applied; secondary and subgroup analyses are therefore regarded as exploratory and hypothesis-generating, and inference is focused on the pre-specified primary composite outcome.

## Results

### Patient demographics and preoperative health

Of 3894 patients, 3421 (88%) received autografts and 473 (12%) received alloplastic materials. Regarding defect size, 2322 (60%) were ≤5 cm and 1572 (40%) were >5 cm. The mean age was 54.1 years, although alloplastic patients were significantly younger on average (51.0 vs. 54.5 years, *p* < 0.001). 2218 (57%) of patients were female, and most (n = 2409, 62%) were classified as ASA III. Alloplastic recipients were more likely to present as emergency cases (n = 27, 12% vs. n = 147, 5.7%, *p* < 0.001) and had higher rates of functional dependency (n = 47, 10% vs. n = 152, 4.5%, *p* < 0.001). The distribution of defects >5 cm was nearly identical between the autograft (n = 1384, 41%) and alloplastic (n = 188, 40%) groups (*p* = 0.81). See Table 1, Supplemental Table S1.

**Table 1:** Patient Characteristics by Cranioplasty Material Type.

Characteristic	Overall N = 3894	Autograft N = 3421	Alloplastic N = 473	p-value^1^
Age (years), Mean (SD)	54.1 (15.7)	54.5 (15.6)	51.0 (16.3)	<0.001
Female sex, n (%)	2218 (57.0%)	1962 (57.4%)	256 (54.1%)	0.20
Race, n (%)				0.12
White	2710 (69.6%)	2357 (68.9%)	353 (74.6%)	
Black	324 (8.3%)	287 (8.4%)	37 (7.8%)	
Asian	130 (3.3%)	118 (3.4%)	12 (2.5%)	
Other	23 (0.6%)	20 (0.6%)	3 (0.6%)	
Unknown	707 (18.2%)	639 (18.7%)	68 (14.4%)	
Hispanic ethnicity, n (%)	121 (3.1%)	102 (3.0%)	19 (4.0%)	0.28
BMI (kg/m²), Mean (SD)	29.2 (6.9)	29.3 (7.0)	28.9 (6.5)	0.277
Obesity (BMI ≥30), n (%)	1512 (38.8%)	1330 (38.9%)	182 (38.5%)	0.907
Diabetes mellitus, n (%)	498 (12.8%)	428 (12.5%)	70 (14.8%)	0.19
Current smoker, n (%)	685 (17.6%)	611 (17.9%)	74 (15.6%)	0.26
Dyspnea, n (%)	113 (2.9%)	107 (3.1%)	6 (1.3%)	0.035
Ventilator dependent, n (%)	66 (1.7%)	51 (1.5%)	15 (3.2%)	0.014
COPD, n (%)	120 (3.1%)	110 (3.2%)	10 (2.1%)	0.25
Ascites, n (%)	2 (0.1%)	2 (0.1%)	0 (0.0%)	>0.99
Congestive heart failure, n (%)	32 (0.8%)	21 (0.6%)	11 (2.3%)	<0.001
Hypertension, n (%)	1491 (38.3%)	1333 (39.0%)	158 (33.4%)	0.023
Acute renal failure, n (%)	5 (0.1%)	3 (0.1%)	2 (0.4%)	0.22
Dialysis, n (%)	9 (0.2%)	8 (0.2%)	1 (0.2%)	>0.99
Disseminated cancer, n (%)	498 (12.8%)	451 (13.2%)	47 (9.9%)	0.056
Chronic steroid use, n (%)	382 (9.8%)	343 (10.0%)	39 (8.2%)	0.26
Bleeding disorder, n (%)	80 (2.1%)	70 (2.0%)	10 (2.1%)	>0.99
Preoperative transfusion, n (%)	20 (0.5%)	17 (0.5%)	3 (0.6%)	0.96
Weight loss >10%, n (%)	49 (1.3%)	45 (1.3%)	4 (0.8%)	0.52
Functional status, n (%)				<0.001
Independent	3637 (94.8%)	3216 (95.5%)	421 (90.0%)	
Partially Dependent	168 (4.4%)	133 (3.9%)	35 (7.5%)	
Totally Dependent	31 (0.8%)	19 (0.6%)	12 (2.6%)	
ASA class, n (%)				0.005
I	67 (1.7%)	65 (1.9%)	2 (0.4%)	
II	1050 (27.0%)	945 (27.6%)	105 (22.2%)	
III	2409 (61.9%)	2094 (61.2%)	315 (66.6%)	
IV	368 (9.5%)	317 (9.3%)	51 (10.8%)	
Operative time (min), Mean (SD)	259.4 (157.8)	260.8 (158.3)	249.1 (154.2)	0.13
Emergency case, n (%)	174 (6.2%)	147 (5.7%)	27 (12.2%)	<0.001
Wound classification, n (%)				0.96
Clean	2586 (91.7%)	2383 (91.7%)	203 (91.9%)	
Clean/Contaminated	145 (5.1%)	133 (5.1%)	12 (5.4%)	
Contaminated	40 (1.4%)	37 (1.4%)	3 (1.4%)	
Dirty/Infected	50 (1.8%)	47 (1.8%)	3 (1.4%)	
Inpatient setting, n (%)	3786 (97.2%)	3328 (97.3%)	458 (96.8%)	0.68
Defect size >5 cm, n (%)	1572 (40.4%)	1384 (40.5%)	188 (39.7%)	0.81

Values: mean (SD) for continuous, n (%) for categorical.

SD = standard deviation; BMI = body mass index; COPD = chronic obstructive pulmonary disease; CHF = congestive heart failure; ASA = American Society of Anesthesiologists.

1 Two Sample *t*-test; Pearson's Chi-squared test.

### Surgical characteristics, Peri- and postoperative outcomes

The overall 30-day complication rate was 22% (n = 852). No significant difference was observed in the unadjusted rates of “any complication” between autograft (22%, n = 745) and alloplastic (23%, n = 107) materials (*p* = 0.72). See Table 2. Alloplastic materials were associated with a significantly higher rate of non-home discharge (28% vs. 20%, *p* = 0.012) but a lower rate of UTIs (n = 2, 0.4% vs. n = 62, 1.8%, *p* = 0.042). When stratifying by both material and size, the autograft >5 cm group exhibited the highest raw complication rate at 25% (n = 352), followed by alloplastic ≤5 cm 24% (n = 68), alloplastic >5 cm 21% (n = 39), and autograft ≤5 cm 19% (n = 393) (*p* < 0.001). See Supplemental Table S2. The most common complication following cranioplasty was bleeding requiring transfusion, which occurred in 8.1% (n = 317) of the total cohort.

**Table 2:** 30-Day Outcomes by Cranioplasty Material Type.

Characteristic	Overall N = 3894	Autograft N = 3421	Alloplastic N = 473	p-value^1^
Any complication, n (%)	852 (21.9%)	745 (21.8%)	107 (22.6%)	0.72
Surgical complication, n (%)	142 (3.6%)	126 (3.7%)	16 (3.4%)	0.84
Any SSI, n (%)	124 (3.2%)	108 (3.2%)	16 (3.4%)	0.90
Superficial SSI, n (%)	49 (1.3%)	44 (1.3%)	5 (1.1%)	0.84
Deep incisional SSI, n (%)	26 (0.7%)	23 (0.7%)	3 (0.6%)	>0.99
Organ/space SSI, n (%)	51 (1.3%)	43 (1.3%)	8 (1.7%)	0.57
Wound dehiscence, n (%)	36 (0.9%)	34 (1.0%)	2 (0.4%)	0.34
Medical complication, n (%)	418 (10.7%)	365 (10.7%)	53 (11.2%)	0.78
Pneumonia, n (%)	101 (2.6%)	87 (2.5%)	14 (3.0%)	0.70
Unplanned reintubation, n (%)	97 (2.5%)	87 (2.5%)	10 (2.1%)	0.69
Pulmonary embolism, n (%)	39 (1.0%)	34 (1.0%)	5 (1.1%)	>0.99
Ventilator >48 h, n (%)	141 (3.6%)	118 (3.4%)	23 (4.9%)	0.16
Venous thromboembolism, n (%)	108 (2.8%)	97 (2.8%)	11 (2.3%)	0.63
Deep vein thrombosis, n (%)	82 (2.1%)	76 (2.2%)	6 (1.3%)	0.24
Urinary tract infection, n (%)	64 (1.6%)	62 (1.8%)	2 (0.4%)	0.042
Stroke/CVA, n (%)	88 (2.3%)	78 (2.3%)	10 (2.1%)	0.95
Cardiac arrest, n (%)	14 (0.4%)	13 (0.4%)	1 (0.2%)	0.87
Myocardial infarction, n (%)	10 (0.3%)	10 (0.3%)	0 (0.0%)	0.49
Bleeding requiring transfusion, n (%)	317 (8.1%)	270 (7.9%)	47 (9.9%)	0.15
Sepsis, n (%)	66 (1.7%)	56 (1.6%)	10 (2.1%)	0.57
Septic shock, n (%)	21 (0.5%)	16 (0.5%)	5 (1.1%)	0.19
Return to OR, n (%)	290 (7.4%)	262 (7.7%)	28 (5.9%)	0.21
Unplanned reoperation, n (%)	286 (7.3%)	258 (7.5%)	28 (5.9%)	0.24
Readmission, n (%)	372 (9.6%)	338 (9.9%)	34 (7.2%)	0.075
Unplanned readmission, n (%)	359 (9.2%)	326 (9.5%)	33 (7.0%)	0.087
30-day mortality, n (%)	63 (1.6%)	52 (1.5%)	11 (2.3%)	0.27
Non-home discharge, n (%)	588 (20.9%)	526 (20.3%)	62 (27.7%)	0.012
Length of stay (days), Mean (SD)	5.4 (12.9)	5.3 (12.6)	5.6 (15.1)	0.68

Values: mean (SD) for continuous, n (%) for categorical.

SSI = surgical site infection; VTE = venous thromboembolism; DVT = deep vein thrombosis; UTI = urinary tract infection; MI = myocardial infarction; CVA = cerebrovascular accident; LOS = length of stay; OR = operating room.

1 Pearson's Chi-squared test; Two Sample *t*-test.

Regarding operative duration, alloplastic implants >5 cm required the shortest mean surgical time (189 min), a nearly 90-minute reduction compared to autografts of the same size (279 min). Operative duration was longer for autograft defects with larger defect size (279 min vs. 248 min, >5 cm vs. ≤5 cm, respectively). Alloplastic reconstructions for defects ≤5 cm were the most time-intensive at 289 min (*p* < 0.001, Supplementary Table S1).

### Temporal trends

Annual case volume grew from 68 in 2011 to a peak of 416 in 2022 (Table 3). Within this timeframe, the percent of alloplastic cases grew from 7.4% (n = 5 of 68) to 35% (n = 144 of 416) (OR 1.21/year; *p* < 0.001). Concurrently, the adjusted risk of any complication decreased by 4.5% annually (OR 0.955; *p* = 0.013), and medical complications declined significantly (OR 0.95/year; *p* < 0.001). Mean length of stay decreased from 8.3 days in 2011 to 3.3 days in 2023.

**Table 3:** Temporal Trends in Cranioplasty: Case Volume, Material Use, and Outcomes (2011–2023).

Year	N	Alloplastic (%)	>5 cm (%)	Mean Age	Any Compl (%)	SSI (%)	Mortality (%)	Non-Home (%)	Mean LOS
2011	68	7.4	42.6	55.1	25.0	5.9	1.5	25	8.3
2012	251	6.0	48.6	55.9	28.3	2.8	1.2	21.9	7.7
2013	250	6.4	41.6	55.3	23.2	2.4	0.8	20.1	7.0
2014	289	4.8	34.9	54.9	19.7	2.8	2.1	21.5	5.9
2015	273	8.8	39.6	54.6	26.7	3.7	1.8	22.3	7.7
2016	400	5.8	39.0	55.3	19.2	2.5	1.8	20.3	6.9
2017	320	5.9	43.4	54.3	21.2	3.8	1.2	19.4	4.4
2018	312	9.9	40.4	55.1	19.9	2.6	1.0	20.3	5.3
2019	306	8.8	49.7	54.4	18.3	2.9	0.7	16.1	5.1
2020	352	13.4	45.7	55.7	25.9	3.7	2.3	22.2	4.3
2021	381	17.1	35.7	52.0	19.4	3.7	1.8	N/A*	3.7
2022	416	34.6	32.5	50.3	22.6	2.9	1.4	N/A*	4.1
2023	276	15.6	37.3	52.7	19.6	4.0	3.3	N/A*	3.3

Values are % unless otherwise specified. LOS = length of stay (days).

⁎ Non-home discharge unavailable for 2021–2023 due to DISCHDEST variable coding change in NSQIP.

### Multivariate logistic regression analysis

On univariable analysis, multiple patient and procedural variables were significantly associated with any 30-day complication, including diabetes (OR 1.56, *p* < 0.001), COPD (OR 1.75, *p* = 0.006), CHF (OR 2.81, *p* = 0.006), hypertension (OR 1.55, *p* < 0.001), bleeding disorder (OR 2.57, *p* < 0.001), disseminated cancer (OR 1.26, *p* = 0.039), and inpatient setting (OR 2.80, *p* < 0.001). Other significant predictors included defect size >5 cm (OR 1.34), steroid use (OR 1.62), preop transfusion (OR 6.72), functional dependence (OR 2.07), ventilator dependence (OR 12.8) and emergency cases (OR 2.57) (all *p* < 0.05). See Supplementary Table S1.

**Fig. 2:**
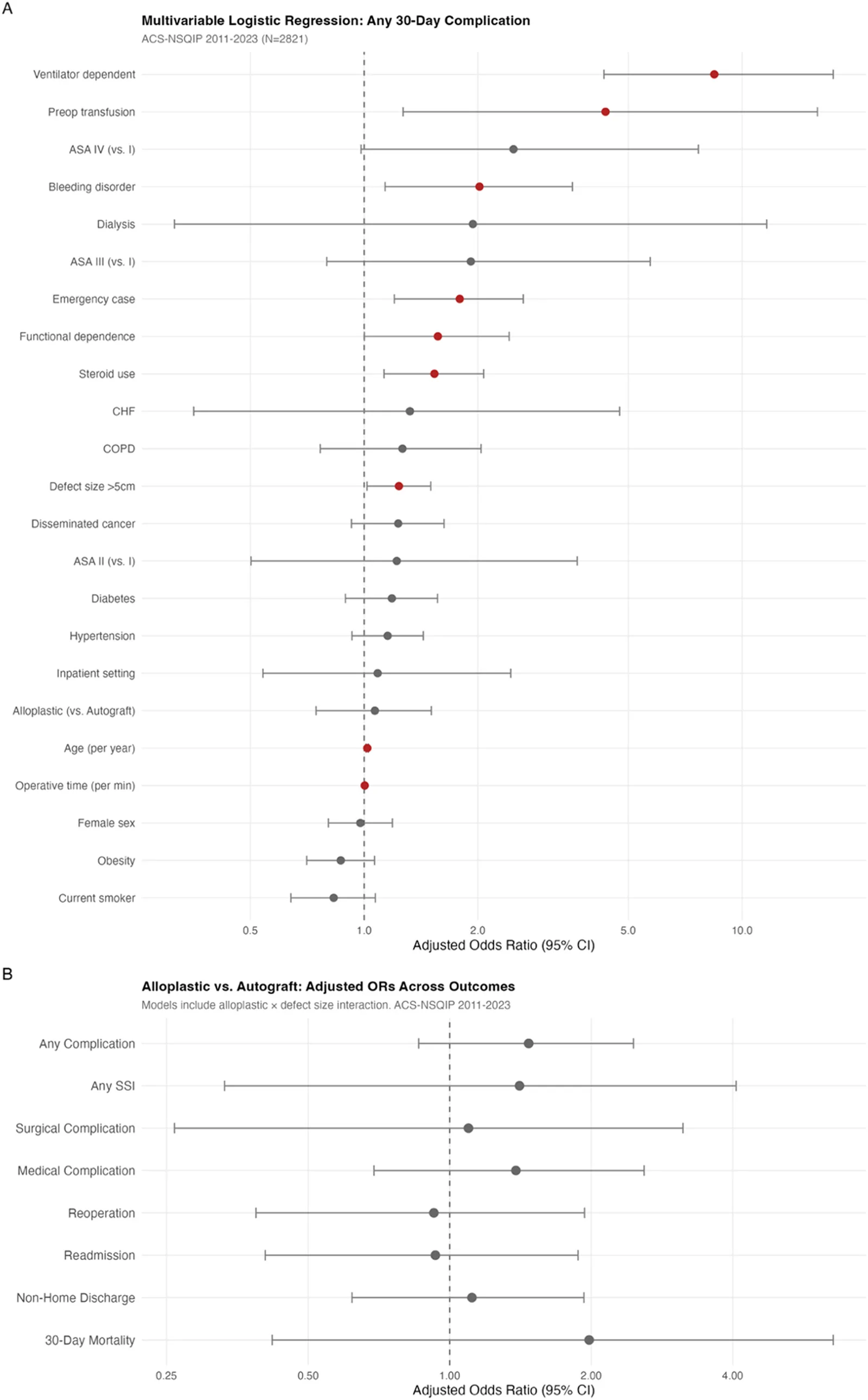
Forest Plots of Multivariable Logistic Regression for 30-Day Complications. (A) Adjusted odds ratios (aORs) with 95% confidence intervals for any 30-day complication from multivariable logistic regression adjusting for 21 covariates (N = 2821 complete cases). Red dots indicate statistically significant predictors (*p* < 0.05). The dashed vertical line represents the null value (OR = 1.0). (B) Adjusted odds ratios of alloplastic material (vs. autograft) across primary and secondary outcomes. Models include an alloplastic × defect size interaction term and adjust for all covariates. None of the secondary outcomes showed a statistically significant difference between material types. ACS-NSQIP, American College of Surgeons National Surgical Quality Improvement Program; CI, confidence interval; ASA, American Society of Anesthesiologists; COPD, chronic obstructive pulmonary disease; CHF, congestive heart failure; SSI, surgical site infection.

In multivariable analysis, nine independent predictors of any 30-day complication were identified (Table 4, Fig. 2A). The strongest independent association with this primary outcome was ventilator dependence (aOR 8.44, 95% CI 4.31–17.43; p < 0.001), followed by a preoperative transfusion (aOR 4.35, 95% CI 1.27–15.82; *p* = 0.020). Additional independent risk factors included emergency case (aOR 1.79, 95% CI 1.20–2.64; *p* = 0.004), bleeding disorders (aOR 2.00; *p* = 0.018), functional dependence (aOR 1.56; *p* = 0.048), chronic steroid use (aOR 1.54; *p* = 0.006), advanced age (aOR 1.02 per year; *p* < 0.001), and increased operative time (aOR 1.003; *p* < 0.001). While a larger defect size >5 cm was an independent predictor of complications (aOR 1.24; *p* = 0.032), the choice of alloplastic material compared to autograft was not a significant predictor of complications (*p* = 0.72) nor were other comorbidities such as diabetes, COPD, or current smoking status in this specific cohort.

**Table 4:** Multivariable Logistic Regression for Any 30-Day Complication ACS-NSQIP 2011–2023 (N = 2821 complete cases) C-statistic (AUC): 0.737.

Characteristic	OR (95% CI)	p-value
Alloplastic (vs. autograft)	1.07 (0.75 to 1.51)	0.715
Defect >5cm	1.24 (1.02 to 1.50)	**0.032**
Age (years)	1.02 (1.01 to 1.03)	**<0.001**
Female sex	0.97 (0.80 to 1.18)	0.76
Obesity (BMI≥30)	0.87 (0.70 to 1.07)	0.176
Diabetes	1.17 (0.88 to 1.54)	0.28
Current smoker	0.83 (0.64 to 1.07)	0.16
Ventilator dependent	8.44 (4.31 to 17.43)	<0.001
COPD	1.26 (0.76 to 2.03)	0.35
CHF	1.14 (0.30 to 4.15)	0.84
Hypertension	1.12 (0.90 to 1.38)	0.32
Dialysis	1.90 (0.31 to 11.3)	0.47
Disseminated cancer	1.25 (0.94 to 1.65)	0.12
Steroid use	1.54 (1.13 to 2.08)	**0.006**
Bleeding disorder	2.00 (1.12 to 3.54)	**0.018**
Preop transfusion	4.35 (1.27 to 15.82)	**0.020**
Functional dependence	1.56 (1.00 to 2.41)	**0.048**
ASA class		
I	—	
II	1.17 (0.48 to 3.53)	0.75
III	1.82 (0.76 to 5.43)	0.22
IV	2.54 (1.00 to 7.82)	0.071
Operative time (min)	1.00 (1.00 to 1.00)	**<0.001**
Emergency case	1.79 (1.20 to 2.64)	**0.004**
Inpatient setting	1.12 (0.56 to 2.53)	0.77

Abbreviations: CI = Confidence Interval, OR = Odds Ratio.

Bold = *p* < 0.05.

Reference: Autograft (material), ≤5 cm (defect), ASA I.

### Interaction and subgroup analysis

No significant interaction was found between material type and defect size (Interaction *p* = 0.151, Supplementary Table S3, Fig. 2B) or ASA risk category (*p* = 0.217, Supplementary Table S4) for any 30-day complication. When stratified by defect size, alloplastic material was not associated with significantly different odds of complications compared to autograft for either small (≤5 cm; *p* = 0.220) or large defects (>5 cm; *p* = 0.521). See Supplementary Figure 1. Similarly, overall complication risk did not differ significantly by material type in either low-risk (*p* = 0.202) or high-risk (*p* = 0.926) patient cohorts (Supplementary Table S4).

However, exploratory analysis identified a significant interaction between material type and ASA classification regarding 30-day readmissions (*p* = 0.040). More specifically, low-risk patients (ASA I-II) had significantly higher odds of 30-day readmission (aOR 2.41, 95% CI 1.04–5.10, *p* = 0.029). No such association between material type and readmission was observed among high-risk (ASA III-IV) patients (*p* = 0.347).

Consistent with the primary analysis, no other secondary outcomes, including SSI, surgical complications, or medical complications, showed significant interactions between material type and clinical strata (all *p* > 0.05). When stratified by size, alloplastic material was not associated with differences in the odds of complications for either small (≤5 cm) or large (>5 cm) defects relative to autograft (Supplementary Table S5).

### Predictive modeling performance

**Fig. 3:**
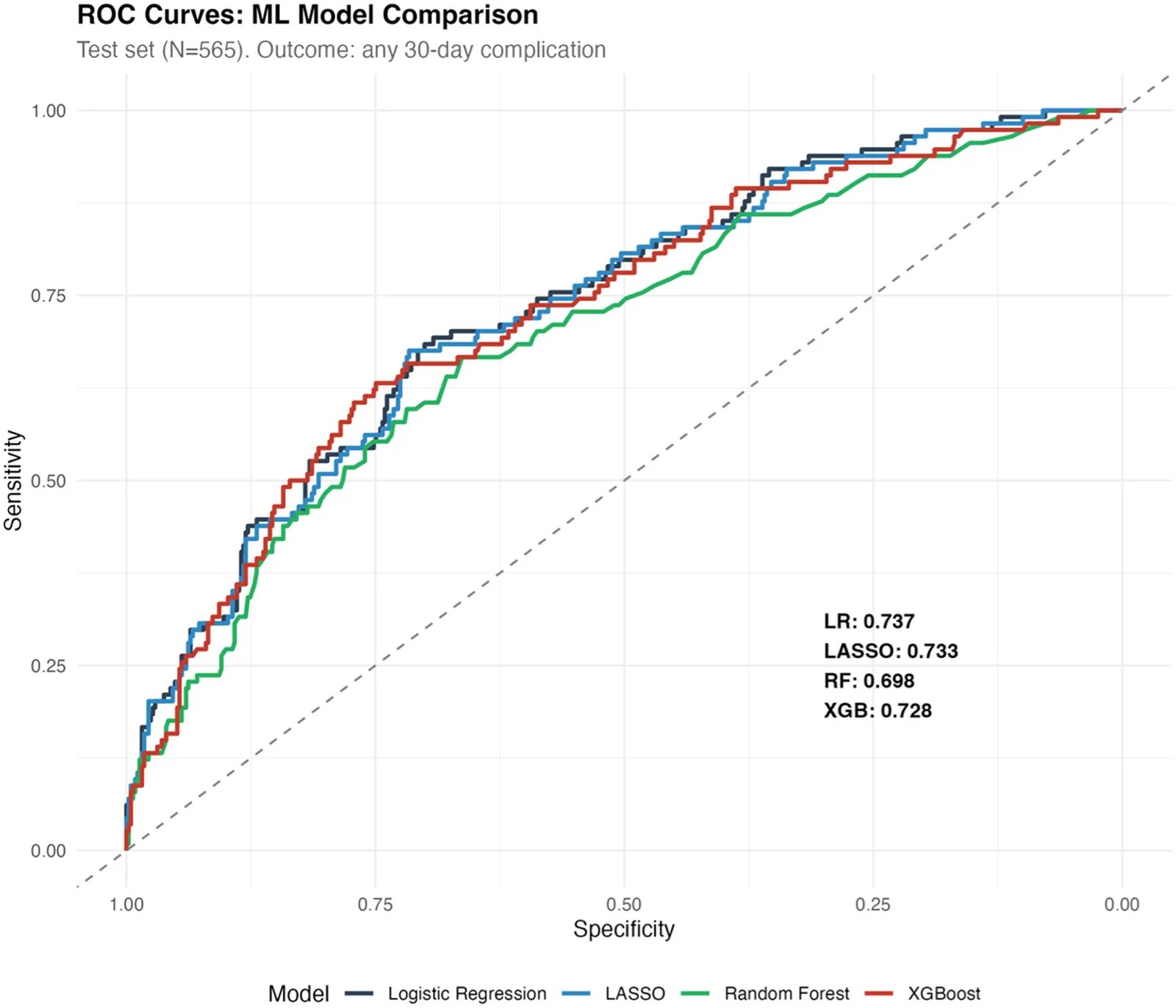
Receiver Operating Characteristic (ROC) Curves for Machine Learning Models.

**Fig. 4:**
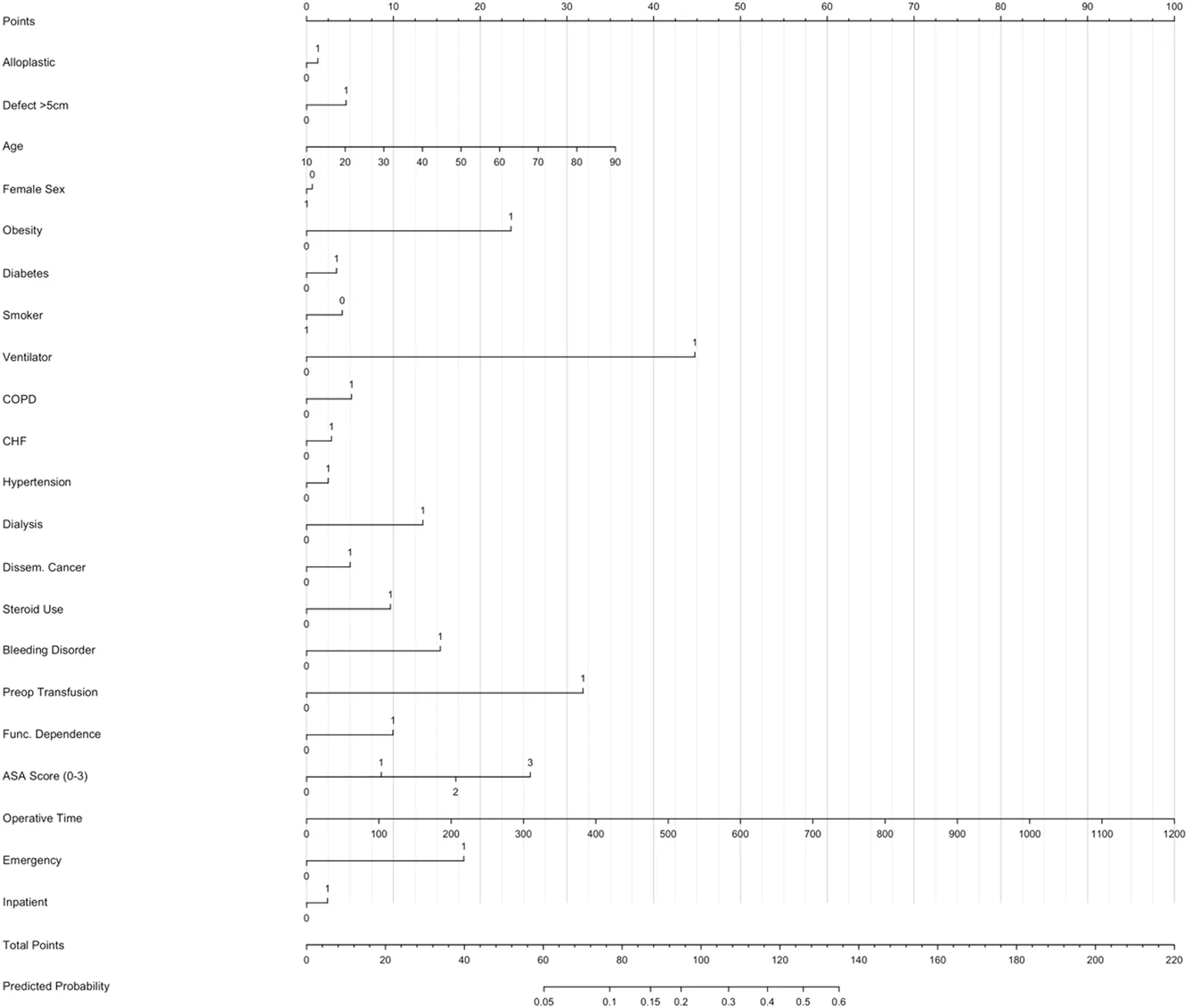
Clinical Nomogram for Predicting 30-Day Complication Risk Following Cranioplasty.

AI-supported machine learning models were trained to predict 30-day complications (Supplementary Table S6, Fig. 3). Logistic Regression and LASSO models achieved the highest discriminative performance with AUCs of 0.737 and 0.733, respectively. XGBoost (AUC 0.728) and Random Forest (AUC 0.698) models demonstrated slightly lower performance. Given that the complex ML algorithms did not outperform logistic regression, this model was selected for the development of a clinical nomogram (Fig. 4).

## Discussion

In the largest multi-institutional analysis of cranioplasty outcomes to date, reconstructive material was not independently associated with 30-day complications, irrespective of defect size or patient risk profile. Instead, outcomes were primarily driven by patient-related factors (e.g., ventilator dependence, steroid use) and procedural characteristics (e.g., emergency status, operative time, defect size). Notably, despite a fivefold increase in alloplastic utilization over 13 years, complication rates declined in parallel, consistent with an improving safety profile for alloplastic cranioplasty though not necessarily caused by it. These findings suggest that outcomes are more strongly determined by host physiology and operative burden than by the intrinsic properties of the reconstructive material. Importantly, however, these conclusions are limited to 30-day perioperative risk. The selection of alloplastic materials in this cohort was non-random and driven by clinical factors including higher rates of emergency presentation and functional dependence, and residual confounding by indication cannot be fully excluded despite multivariable adjustment.

Although autologous bone has been the historical standard for cranioplasty, our multivariable analysis demonstrated that alloplastic materials are associated with a comparable short-term perioperative safety profile with an adjusted odds ratio (aOR) of 1.07 for any complication. This null finding stands in contrast with prior literature performed on smaller patient cohorts. Mukherjee et al. (n = 174) demonstrated that patients with larger skull defects repaired with titanium cranioplasties had elevated complication rates (26%), with infection as a primary driver for nearly 70% of plate removals.^13^ Similarly, a 2025 multi-center study by Yang et al. (n = 191) found that titanium use was an independent risk factor for major complications and new-onset seizures in trauma patients, while defect size itself was not significant.^20^ However, these studies employed extended follow-up periods beyond 30 days, capturing late complications such as delayed infection and implant extrusion that fall outside the NSQIP observation window — a distinction that likely explains the higher absolute complication rates reported compared to our 30-day data. Furthermore, our interaction analysis addresses a key gap in the literature by evaluating whether the performance of synthetic materials varies with increasing defect size. No significant interaction was observed, suggesting that, in the short-term, alloplastic materials maintain consistent performance across defect sizes. These findings indicate that alloplastic reconstruction may not only be suitable for smaller defects but also represents a reliable option for larger defects, where autologous bone may be insufficient. It should be noted, however, that several prior studies reporting higher complication rates for alloplastic materials employed longer follow-up periods, capturing late infections, implant exposure, and hardware failure that fall outside the 30-day NSQIP window. The apparent discrepancy between our findings and prior literature may therefore partly reflect this methodological difference rather than a true contradiction, and should not be interpreted as evidence that alloplastic materials are unconditionally equivalent to autograft across all timepoints.

While material choice was neutral, a defect size greater than 5 cm was identified as an independent predictor of complications (aOR 1.24). Interestingly, alloplastic materials offered a significant technical advantage in these larger cases, with approximately 90 min of operative time saved compared to autografts (189 min vs 279 min). This efficiency is likely attributable to the use of pre-fabricated or custom 3D-printed implants eliminating the need for intraoperative bone contouring or donor site harvest.^8^^,^^21^ Given that increased operative time itself was confirmed as an independent risk factor for complications, the time saved from alloplastic material utilization may actually be safer for complex reconstructions, at least in terms of short-term complications. Shorter procedures likely mitigate cumulative anesthetic exposure, blood loss, and inflammatory response overall, all which disproportionately impact high-risk patients.^22^ Notably, alloplastic reconstruction for small defects (≤5 cm) was associated with the longest mean operative time across all four groups at 289 min, exceeding even autograft reconstruction for large defects. This counterintuitive finding most likely reflects differences in case complexity and patient selection within this subgroup rather than a true material-related effect, and should be interpreted with caution given the limited number of alloplastic cases in this stratum.

Our observation that low-risk (ASA I-II) alloplastic recipients faced higher readmission rates (aOR 2.41) warrants careful interpretation. This finding should be viewed with caution: the low-risk alloplastic subgroup was small, the analysis was post-hoc and exploratory in nature, and no correction for multiple comparisons was applied. Prior literature suggests that while synthetic materials improve intraoperative efficiency, these gains do not consistently translate into reduced postoperative utilization. Mozaffari et al. demonstrated that an apparent reduction in length of stay with alloplastic implants was no longer significant after adjustment for patient frailty.^23^ Khalid et al. reported modest and material-specific reductions in hospitalization duration, while Gilardino et al. reported decreased ICU utilization with synthetic implants.^12^^,^^21^ Within this context, one potential explanation is that the perceived efficiency and technical ease of alloplastic procedures may lend themselves to more streamlined perioperative pathways in otherwise healthy patients, potentially shifting rather than eliminating postoperative risk.^11^ Alternatively, this finding could reflect differences in unmeasured factors such as outpatient follow-up adherence or early wound-related concerns not captured within NSQIP. Either way, this finding should be considered hypothesis-generating only and requires prospective validation before informing clinical practice.

Our results support a more individualized, context-driven approach to material selection. Although debates have historically centered on the immunologic and infectious profiles of autologous versus synthetic materials, our data suggest that these differences may be clinically subordinate to baseline physiologic reserve and intraoperative stress.^2^^,^^24^^,^^25^ This may explain why material type did not emerge as significant even after risk adjustment and interaction testing. For example, in patients with limited physiologic reserve or anticipated prolonged operative times, the use of prefabricated alloplastic implants may confer indirect benefit through procedural efficiency, even if the material itself is not associated with reduced complication risk. Indeed, as some experts argue that “no ideal material currently exists,” the decision-making process is shifting toward a more individualized approach.^26^ In the absence of a single, clearly superior material, synthetic options now represent viable, and often more efficient, alternatives for modern cranial reconstruction.

Notably, over the 13-year study period, we observed a marked shift in surgical practice. Alloplastic adoption increased nearly five-fold to a peak of 35% in 2022. This surge in synthetic material use coincided with an improving safety profile, with adjusted complication risks decreasing by 4.5% annually. These parallel trends are consistent with an improving safety profile for alloplastic cranioplasty over time, though causality cannot be inferred. The concurrent decline in complication rates most likely reflects broader advances in perioperative care, surgical experience, and institutional protocols—including the implementation of perioperative bundles and standardized wound healing protocols—rather than a direct effect of material adoption.^27^^,^^28^

Nine independent predictors of 30-day morbidity were identified, with the strongest related to preoperative physiologic reserve. This included ventilator dependence (aOR 8.44) and preoperative transfusion (aOR 4.35). Following comparison with several machine learning architectures, standard logistic regression outperformed these more complex models in terms of discriminative power (AUC 0.737). Consequently, we developed a validated clinical nomogram based on this logistic model to provide surgeons with a reliable tool for individualized risk stratification. Although recent surgical literature has seen a surge in AI-based ML applications aiming for superior predictive power, our analysis demonstrates that increased model complexity does not necessarily translate to improved clinical utility.^29^, ^30^, ^31^ Conventional logistic regression (AUC 0.737) achieved the highest discriminative performance, higher than more complex architectures such as LASSO, XGBoost, and Random Forest. These black box algorithms raise significant concerns regarding accountability, the perpetuation of existing biases, and legal liability.^32^^,^^33^ The ability to explain is among the physician behaviors most strongly associated with patient trust across all patient groups regardless of sex, age, or length of relationship with the physician.^34^

## Limitations

This study should be interpreted in light of several limitations. First, the retrospective nature of this study introduces the potential for selection bias and unmeasured confounding. While multivariable adjustment was performed using a large set of clinically relevant variables, there remains a potential for residual confounding in variables not captured explicitly within the ACS-NSQIP dataset. Furthermore, the decision to use alloplastic versus autologous reconstruction is inherently non-random and driven by clinical factors that may not be fully captured within NSQIP. Patients selected for alloplastic reconstruction in this cohort were more likely to present as emergency cases, exhibit functional dependence, and carry a higher burden of cardiopulmonary comorbidity. Although multivariable adjustment was performed, residual confounding by indication cannot be excluded, and the observed equivalence in complication rates between material types should be interpreted as reflecting adjusted short-term perioperative risk rather than demonstrated material equivalence in an unselected population. Furthermore, alloplastic cases were disproportionately represented among patients excluded from the multivariable analysis due to missing covariate data (23.5% vs. 7.8% of complete cases, *p* < 0.001), driven by the temporal coincidence of NSQIP variable discontinuation and increasing alloplastic adoption; this may result in a modest underestimation of absolute complication rates in the multivariable model, though the primary null finding for material type is unlikely to be affected. In particular, the underlying indication for cranioplasty—such as trauma, tumor resection, infection, or decompression for ischemic or hemorrhagic stroke—is not available as a discrete, reliably populated field within the ACS-NSQIP Participant Use Files. Although diagnosis (ICD) codes can in principle be queried in some data environments, within the Participant Use Files they are inconsistently captured and not reliably linked to individual cases, which precludes formal adjustment for indication. Because the underlying diagnosis may plausibly influence both the choice of reconstructive material and the risk of complications, this omission represents an additional potential source of residual confounding. Second, the NSQIP database is limited to 30-day postoperative outcomes. This represents a critical constraint when evaluating alloplastic cranioplasty materials, as complication profiles among alloplastic subtypes diverge substantially beyond the perioperative window. Late-stage complications that may take months or years to manifest—such as delayed implant infections, hardware extrusion, and long-term autologous bone resorption—are not captured by NSQIP, and the present analysis likely underestimates the true long-term complication rate. Meta-analysis with minimum 12-month follow-up has demonstrated pooled all-cause failure rates of 6.3% for PEEK, 11% for titanium, and 13% for PMMA, with overall complication rates of 19%, 26%, 28% respectively and 2.3% for porous polyethylene.^9^^,^^24^ Material-specific long-term complications—including titanium implant exposure, PMMA-associated infection rates approaching 15% at extended follow-up, PEEK-related subcutaneous effusion occurring in up to 33% of cases and porous polyethylene implant exposure rates of 15% in large defects (particularly when tissue expansion is employed)—manifest well beyond the 30-day capture window of NSQIP.^35^, ^36^, ^37^ Consequently, our findings should be interpreted strictly as reflecting short-term perioperative risk equivalence and cannot be extrapolated to long-term material performance, where clinically meaningful differences between alloplastic subtypes are well documented. This limitation is further compounded by the fact that NSQIP does not capture granular data on specific alloplastic subtypes, which themselves are known to be associated with varying risk and benefit profiles.^1^^,^^6^^,^^38^ The present study therefore addresses a single, early timepoint in a complication trajectory that extends across months to years, and prospective studies with long-term follow-up stratified by material subtype are needed to fully characterize the comparative safety profiles of modern alloplastic options. Third, defect size was derived from procedural CPT code classification rather than direct intraoperative measurement. As such, it represents a billing-based surrogate that is subject to coder interpretation and potential misclassification. True anatomical defect dimensions were not available within the NSQIP dataset, and the magnitude and directional impact of any misclassification on the interaction analysis cannot be determined. Fourth, the autograft category as defined by CPT codes 62,146 and 62,147 encompasses autologous bone cranioplasty broadly and does not distinguish between stored bone flap replacement, split-thickness calvaria, or other autologous bone sources. While stored bone flap replacement is presumed to represent the majority of cases in this cohort, the contribution of other autologous bone sources cannot be quantified within NSQIP, and their potentially differing complication profiles introduce heterogeneity within the autograft group. Fifth, NSQIP does not capture data regarding aesthetic symmetry, patient satisfaction, or long-term neurological recovery. Cranial vault aesthetics significantly impact patient satisfaction following cranioplasty, with contour irregularities and appearance concerns strongly associated with psychological distress and reduced quality of life.^39^^,^^40^ Finally, although NSQIP is multi-institutional, the participating hospitals may be more representative of larger academic centers than smaller community or non-academic facilities. Therefore, the generalizability of these findings to all practice settings may be limited. A further consideration is that the primary composite “any complication” endpoint aggregates events of markedly heterogeneous clinical significance, ranging from urinary tract infection and transfusion to stroke, myocardial infarction, and death, and likewise incorporates medical events (such as reintubation or urinary tract infection) that are less directly attributable to the reconstructive material than surgical outcomes. Pooling events of unequal severity and differing mechanistic relevance may dilute or obscure material-specific effects on the complications of greatest clinical importance; for this reason the individual components are reported separately in Table 2, and the composite endpoint should be interpreted with this heterogeneity in mind. Lastly, no formal sensitivity analyses were performed; this represents a limitation, and future studies should consider analyses restricted to elective cases or excluding emergency presentations to assess the robustness of these findings.

## Conclusion

Material type is not independently associated with adjusted 30-day complications following cranioplasty, regardless of defect size or baseline patient risk. For large defects (>5 cm), alloplastic reconstruction was associated with a 90-minute reduction in operative time, likely reflecting use of prefabricated implants, while defect size itself remained an independent predictor of morbidity. Nine variables independently predicted complications, with the strongest effects observed for ventilator dependence, and preoperative transfusion. Over the study period, a significant five-fold increase in alloplastic adoption coincided with a 4.5% annual decrease in adjusted complication risk, suggesting that synthetic materials are increasingly integrated into safe, modern surgical protocols. Logistic regression outperformed more complex machine learning models, enabling development of an interpretable nomogram for individualized risk stratification that prioritizes patient physiology and procedural complexity over material selection. These findings support the use of alloplastic techniques as a safe and efficient alternative for cranial reconstruction in the short term. As these conclusions are limited to 30-day perioperative outcomes, prospective comparative studies with extended follow-up stratified by alloplastic subtype are warranted to fully characterize long-term material performance and guide evidence-based material selection.

## Funding source

The authors received no financial support for the research, authorship, or publication of this article. The ACS-NSQIP and the hospitals participating in ACS-NSQIP are the source of the data used herein; they have not verified and are not responsible for the statistical validity of the data analysis or the conclusions derived by the authors.

## Ethical approval

Ethical approval was not required because this study used a retrospective analysis of the de-identified ACS-NSQIP database, which contains anonymized patient data and is therefore exempt from institutional review board oversight.

## Conflicts of interest

The authors declare no conflicts of interest.

## Appendix Supplementary materials

Image, application 1

Image, application 2

Image, application 3
