# Supplementary material for: Material type versus defect size in adult cranioplasty: a multi-institutional analysis of 30-day outcomes and risk prediction modeling
Source: JPRAS Open. 2026 Aug 19;52:213–25. doi: 10.1016/j.jpra.2026.08.024 (PMC13571319; doi:10.1016/j.jpra.2026.08.024)
Supplement: Supplementary file 2 [file mmc2.docx]

# STROBE Checklist — Cohort Studies

| Item No | Recommendation | Where addressed in manuscript / Author action required |
| --- | --- | --- |
| TITLE AND ABSTRACT | | |
| 1a | Indicate the study's design with a commonly used term in the title or the abstract | Abstract; Section 2.1 ("retrospective cohort analysis") |
| 1b | Provide in the abstract an informative and balanced summary of what was done and what was found | Abstract (Background, Methods, Results, Conclusion) |
| INTRODUCTION | | |
| 2 | Explain the scientific background and rationale for the investigation being reported | Section 1 (Background) |
| 3 | State specific objectives, including any prespecified hypotheses | Section 1, final paragraph (objectives i–iv explicitly stated) |
| METHODS | | |
| 4 | Present key elements of study design early in the paper | Section 2.1 and Abstract |
| 5 | Describe the setting, locations, and relevant dates, including periods of recruitment, exposure, follow-up, and data collection | Section 2.1 Data Source |
| 6* | Give the eligibility criteria, and the sources and methods of selection of participants. Describe methods of follow-up | Section 2.2 (Patient Selection); Figure 1 (flow diagram) |
| 7 | Clearly define all outcomes, exposures, predictors, potential confounders, and effect modifiers. Give diagnostic criteria, if applicable | Section 2.3 (Variable Extraction); primary/secondary outcomes defined |
| 8* | For each variable of interest, give sources of data and details of methods of assessment. Describe comparability of assessment methods if there is more than one group | Section 2.3 (CPT code-based classification for material type and defect size) |
| 9 | Describe any efforts to address potential sources of bias | Section 2.4 (multivariable adjustment for 21 covariates; interaction terms); Section 5 (Limitations) |
| 10 | Explain how the study size was arrived at | Section 2.4 Statistical Analysis |
| 11 | Explain how quantitative variables were handled in the analysis. If applicable, describe which groupings were chosen and why | Section 2.3 (defect size ≤5 cm vs. >5 cm defined by CPT codes; ASA I–II vs. III–IV groupings explained in Section 2.4) |
| 12* | Describe all statistical methods, including those used to control for confounding | Section 2.4 (Statistical Analysis) — multivariable logistic regression, LASSO, XGBoost, Random Forest, interaction terms, Cochran-Armitage trend tests |
| 13 | Describe any methods used to examine subgroups and interactions | Section 2.4 (multiplicative interaction terms; stratified analyses by defect size and ASA class) |
| 14 | Explain how missing data were addressed | Section 2.4 (complete-case analysis; 2,821/3,894 patients [72.4%] had complete data; reported explicitly) |
| 15* | If applicable, explain how loss to follow-up was addressed | Section 2.4 Statistical Analysis. ACS-NSQIP captures structured 30-day outcomes for all cases; loss to follow-up is not applicable to this administrative database. |
| 16 | Describe any sensitivity analyses | Section 5 Limitations |
| RESULTS | | |
| 17* | Report numbers of individuals at each stage of study (eligible, examined, confirmed, included, completing follow-up, analysed) | Figure 1 (flow diagram); Section 3.1 (N=3,894 final cohort; exclusions described) |
| 18* | Give characteristics of study participants (demographic, clinical, social) and information on exposures and potential confounders | Table 1 (by material type); Supplementary Table S1 (by material and defect size) |
| 19* | Report numbers of outcome events or summary measures over time | Table 2 (30-day outcomes by material); Table 3 (temporal trends 2011–2023); Section 3.2–3.3 |
| 20* | Give unadjusted and, if applicable, confounder-adjusted estimates and their precision (e.g., 95% CI). Make clear which confounders were adjusted for and why they were included | Supplementary Table S1 (unadjusted); Table 4 (aORs with 95% CIs); 21 covariates listed in Section 2.4 |
| 21 | Report other analyses done (subgroups, interactions, sensitivity analyses) | Section 3.5 (interaction and subgroup analyses); Section 3.6 (predictive modelling, AUC comparisons) |
| DISCUSSION | | |
| 22 | Summarise the key results with reference to study objectives | Section 4 (Discussion), opening paragraph |
| 23 | Discuss limitations of the study, taking into account sources of potential bias or imprecision. Discuss both direction and magnitude of any potential bias | Section 5 (Limitations) — six limitations discussed including selection bias, 30-day window, CPT misclassification, autograft heterogeneity, missing patient-reported outcomes, generalisability |
| 24 | Give a cautious overall interpretation of results considering objectives, limitations, multiplicity of analyses, results from similar studies, and other relevant evidence | Section 4 (Discussion) and Section 6 (Conclusion) |
| 25 | Discuss the generalisability (external validity) of the study results | Section 5, final paragraph (NSQIP hospital representativeness; academic vs. community settings) |
| OTHER INFORMATION | | |
| 26 | Give the source of funding and the role of the funders for the present study and, if applicable, for the original study on which the present article is based | Section 7 Funding and Acknowledgements |

*Reference: von Elm E, Altman DG, Egger M, Pocock SJ, Gøtzsche PC, Vandenbroucke JP; STROBE Initiative. The Strengthening the Reporting of Observational Studies in Epidemiology (STROBE) statement: guidelines for reporting observational studies. Lancet. 2007;370(9596):1453–1457.*
