# Supplementary material for: Material type versus defect size in adult cranioplasty: a multi-institutional analysis of 30-day outcomes and risk prediction modeling
Source: JPRAS Open. 2026 Aug 19;52:213–25. doi: 10.1016/j.jpra.2026.08.024 (PMC13571319; doi:10.1016/j.jpra.2026.08.024)
Supplement: Supplementary file 3 [file mmc3.docx]

# **Table S1**. Patient Characteristics by Material Type and Defect Size

| **Characteristic** | **Overall  N = 3,894** | **Autograft ≤5cm  N = 2,037** | **Autograft >5cm  N = 1,384** | **Alloplastic ≤5cm  N = 285** | **Alloplastic >5cm  N = 188** | **p-value^1^** |
| --- | --- | --- | --- | --- | --- | --- |
| Age (years), Mean (SD) | 54.1 (15.7) | 54.1 (15.6) | 55.2 (15.6) | 48.1 (16.3) | 55.4 (15.3) | <0.001 |
| Female sex, n (%) | 2,218 (57.0%) | 1,159 (56.9%) | 803 (58.0%) | 153 (53.7%) | 103 (54.8%) | 0.52 |
| Race, n (%) |  |  |  |  |  | <0.001 |
| White | 2,710 (69.6%) | 1,402 (68.8%) | 955 (69.0%) | 214 (75.1%) | 139 (73.9%) |  |
| Black | 324 (8.3%) | 135 (6.6%) | 152 (11.0%) | 23 (8.1%) | 14 (7.4%) |  |
| Asian | 130 (3.3%) | 71 (3.5%) | 47 (3.4%) | 4 (1.4%) | 8 (4.3%) |  |
| Other | 23 (0.6%) | 9 (0.4%) | 11 (0.8%) | 2 (0.7%) | 1 (0.5%) |  |
| Unknown | 707 (18.2%) | 420 (20.6%) | 219 (15.8%) | 42 (14.7%) | 26 (13.8%) |  |
| Hispanic ethnicity, n (%) | 121 (3.1%) | 57 (2.8%) | 45 (3.3%) | 11 (3.9%) | 8 (4.3%) | 0.55 |
| BMI (kg/m²), Mean (SD) | 29.2 (6.9) | 29.2 (6.8) | 29.4 (7.2) | 28.8 (6.2) | 29.1 (6.9) | 0.542 |
| Obesity (BMI ≥30), n (%) | 1,512 (38.8%) | 776 (38.1%) | 554 (40.0%) | 119 (41.8%) | 63 (33.5%) | 0.459 |
| Diabetes mellitus, n (%) | 498 (12.8%) | 242 (11.9%) | 186 (13.4%) | 36 (12.6%) | 34 (18.1%) | 0.080 |
| Current smoker, n (%) | 685 (17.6%) | 360 (17.7%) | 251 (18.1%) | 39 (13.7%) | 35 (18.6%) | 0.33 |
| Dyspnea, n (%) | 113 (2.9%) | 68 (3.3%) | 39 (2.8%) | 3 (1.1%) | 3 (1.6%) | 0.11 |
| Ventilator dependent, n (%) | 66 (1.7%) | 28 (1.4%) | 23 (1.7%) | 8 (2.8%) | 7 (3.7%) | 0.046 |
| COPD, n (%) | 120 (3.1%) | 61 (3.0%) | 49 (3.5%) | 2 (0.7%) | 8 (4.3%) | 0.063 |
| Ascites, n (%) | 2 (0.1%) | 1 (0.0%) | 1 (0.1%) | 0 (0.0%) | 0 (0.0%) | 0.95 |
| Congestive heart failure, n (%) | 32 (0.8%) | 12 (0.6%) | 9 (0.7%) | 6 (2.1%) | 5 (2.7%) | 0.002 |
| Hypertension, n (%) | 1,491 (38.3%) | 774 (38.0%) | 559 (40.4%) | 78 (27.4%) | 80 (42.6%) | <0.001 |
| Acute renal failure, n (%) | 5 (0.1%) | 3 (0.1%) | 0 (0.0%) | 1 (0.4%) | 1 (0.5%) | 0.15 |
| Dialysis, n (%) | 9 (0.2%) | 5 (0.2%) | 3 (0.2%) | 1 (0.4%) | 0 (0.0%) | 0.89 |
| Disseminated cancer, n (%) | 498 (12.8%) | 285 (14.0%) | 166 (12.0%) | 24 (8.4%) | 23 (12.2%) | 0.039 |
| Chronic steroid use, n (%) | 382 (9.8%) | 219 (10.8%) | 124 (9.0%) | 20 (7.0%) | 19 (10.1%) | 0.13 |
| Bleeding disorder, n (%) | 80 (2.1%) | 41 (2.0%) | 29 (2.1%) | 3 (1.1%) | 7 (3.7%) | 0.26 |
| Preoperative transfusion, n (%) | 20 (0.5%) | 7 (0.3%) | 10 (0.7%) | 1 (0.4%) | 2 (1.1%) | 0.31 |
| Weight loss >10%, n (%) | 49 (1.3%) | 25 (1.2%) | 20 (1.4%) | 0 (0.0%) | 4 (2.1%) | 0.16 |
| Functional status, n (%) |  |  |  |  |  | <0.001 |
| Independent | 3,637 (94.8%) | 1,932 (96.0%) | 1,284 (94.8%) | 251 (88.7%) | 170 (91.9%) |  |
| Partially Dependent | 168 (4.4%) | 75 (3.7%) | 58 (4.3%) | 24 (8.5%) | 11 (5.9%) |  |
| Totally Dependent | 31 (0.8%) | 6 (0.3%) | 13 (1.0%) | 8 (2.8%) | 4 (2.2%) |  |
| ASA class, n (%) |  |  |  |  |  | <0.001 |
| I | 67 (1.7%) | 41 (2.0%) | 24 (1.7%) | 1 (0.4%) | 1 (0.5%) |  |
| II | 1,050 (27.0%) | 588 (28.9%) | 357 (25.8%) | 57 (20.0%) | 48 (25.5%) |  |
| III | 2,409 (61.9%) | 1,211 (59.5%) | 883 (63.8%) | 211 (74.0%) | 104 (55.3%) |  |
| IV | 368 (9.5%) | 197 (9.7%) | 120 (8.7%) | 16 (5.6%) | 35 (18.6%) |  |
| Operative time (min), Mean (SD) | 259.4 (157.8) | 248.1 (153.9) | 279.4 (162.7) | 288.7 (158.3) | 189.1 (126.3) | <0.001 |
| Emergency case, n (%) | 174 (6.2%) | 93 (6.0%) | 54 (5.1%) | 11 (12.9%) | 16 (11.8%) | <0.001 |
| Wound classification, n (%) |  |  |  |  |  | 0.43 |
| Clean | 2,586 (91.7%) | 1,426 (92.7%) | 957 (90.1%) | 78 (91.8%) | 125 (91.9%) |  |
| Clean/Contaminated | 145 (5.1%) | 72 (4.7%) | 61 (5.7%) | 4 (4.7%) | 8 (5.9%) |  |
| Contaminated | 40 (1.4%) | 20 (1.3%) | 17 (1.6%) | 2 (2.4%) | 1 (0.7%) |  |
| Dirty/Infected | 50 (1.8%) | 20 (1.3%) | 27 (2.5%) | 1 (1.2%) | 2 (1.5%) |  |
| Inpatient setting, n (%) | 3,786 (97.2%) | 1,965 (96.5%) | 1,363 (98.5%) | 278 (97.5%) | 180 (95.7%) | 0.003 |
| ^1^One-way analysis of means; Pearson's Chi-squared test | | | | | | |

Values: mean (SD) for continuous, n (%) for categorical. SSI = surgical site infection; VTE = venous thromboembolism; DVT = deep vein thrombosis; UTI = urinary tract infection; MI = myocardial infarction; CVA = cerebrovascular accident; LOS = length of stay; OR = operating room.

# **Table S2**. 30-Day Outcomes by Material Type and Defect Size

| **Characteristic** | **Overall  N = 3,894** | **Autograft ≤5cm  N = 2,037** | **Autograft >5cm  N = 1,384** | **Alloplastic ≤5cm  N = 285** | **Alloplastic >5cm  N = 188** | **p-value^1^** |
| --- | --- | --- | --- | --- | --- | --- |
| Any complication, n (%) | 852 (21.9%) | 393 (19.3%) | 352 (25.4%) | 68 (23.9%) | 39 (20.7%) | <0.001 |
| Surgical complication, n (%) | 142 (3.6%) | 62 (3.0%) | 64 (4.6%) | 8 (2.8%) | 8 (4.3%) | 0.084 |
| Any SSI, n (%) | 124 (3.2%) | 51 (2.5%) | 57 (4.1%) | 8 (2.8%) | 8 (4.3%) | 0.050 |
| Superficial SSI, n (%) | 49 (1.3%) | 19 (0.9%) | 25 (1.8%) | 2 (0.7%) | 3 (1.6%) | 0.11 |
| Deep incisional SSI, n (%) | 26 (0.7%) | 8 (0.4%) | 15 (1.1%) | 0 (0.0%) | 3 (1.6%) | 0.016 |
| Organ/space SSI, n (%) | 51 (1.3%) | 25 (1.2%) | 18 (1.3%) | 6 (2.1%) | 2 (1.1%) | 0.66 |
| Wound dehiscence, n (%) | 36 (0.9%) | 16 (0.8%) | 18 (1.3%) | 1 (0.4%) | 1 (0.5%) | 0.27 |
| Medical complication, n (%) | 418 (10.7%) | 201 (9.9%) | 164 (11.8%) | 31 (10.9%) | 22 (11.7%) | 0.31 |
| Pneumonia, n (%) | 101 (2.6%) | 53 (2.6%) | 34 (2.5%) | 7 (2.5%) | 7 (3.7%) | 0.78 |
| Unplanned reintubation, n (%) | 97 (2.5%) | 48 (2.4%) | 39 (2.8%) | 5 (1.8%) | 5 (2.7%) | 0.70 |
| Pulmonary embolism, n (%) | 39 (1.0%) | 14 (0.7%) | 20 (1.4%) | 4 (1.4%) | 1 (0.5%) | 0.13 |
| Ventilator >48h, n (%) | 141 (3.6%) | 65 (3.2%) | 53 (3.8%) | 14 (4.9%) | 9 (4.8%) | 0.34 |
| Venous thromboembolism, n (%) | 108 (2.8%) | 48 (2.4%) | 49 (3.5%) | 7 (2.5%) | 4 (2.1%) | 0.19 |
| Deep vein thrombosis, n (%) | 82 (2.1%) | 39 (1.9%) | 37 (2.7%) | 3 (1.1%) | 3 (1.6%) | 0.23 |
| Urinary tract infection, n (%) | 64 (1.6%) | 38 (1.9%) | 24 (1.7%) | 0 (0.0%) | 2 (1.1%) | 0.12 |
| Stroke/CVA, n (%) | 88 (2.3%) | 47 (2.3%) | 31 (2.2%) | 6 (2.1%) | 4 (2.1%) | >0.99 |
| Cardiac arrest, n (%) | 14 (0.4%) | 5 (0.2%) | 8 (0.6%) | 1 (0.4%) | 0 (0.0%) | 0.35 |
| Myocardial infarction, n (%) | 10 (0.3%) | 3 (0.1%) | 7 (0.5%) | 0 (0.0%) | 0 (0.0%) | 0.14 |
| Bleeding requiring transfusion, n (%) | 317 (8.1%) | 123 (6.0%) | 147 (10.6%) | 36 (12.6%) | 11 (5.9%) | <0.001 |
| Sepsis, n (%) | 66 (1.7%) | 34 (1.7%) | 22 (1.6%) | 6 (2.1%) | 4 (2.1%) | 0.90 |
| Septic shock, n (%) | 21 (0.5%) | 9 (0.4%) | 7 (0.5%) | 2 (0.7%) | 3 (1.6%) | 0.22 |
| Return to OR, n (%) | 290 (7.4%) | 143 (7.0%) | 119 (8.6%) | 16 (5.6%) | 12 (6.4%) | 0.18 |
| Unplanned reoperation, n (%) | 286 (7.3%) | 142 (7.0%) | 116 (8.4%) | 16 (5.6%) | 12 (6.4%) | 0.25 |
| Readmission, n (%) | 372 (9.6%) | 195 (9.6%) | 143 (10.3%) | 16 (5.6%) | 18 (9.6%) | 0.11 |
| Unplanned readmission, n (%) | 359 (9.2%) | 189 (9.3%) | 137 (9.9%) | 15 (5.3%) | 18 (9.6%) | 0.11 |
| 30-day mortality, n (%) | 63 (1.6%) | 31 (1.5%) | 21 (1.5%) | 6 (2.1%) | 5 (2.7%) | 0.59 |
| Non-home discharge, n (%) | 588 (20.9%) | 284 (18.5%) | 242 (22.9%) | 24 (27.6%) | 38 (27.7%) | 0.003 |
| Length of stay (days), Mean (SD) | 5.4 (12.9) | 5.1 (12.3) | 5.7 (13.0) | 4.9 (15.2) | 6.6 (15.1) | 0.32 |
| ^1^Pearson's Chi-squared test; One-way analysis of means | | | | | | |

Values: mean (SD) for continuous, n (%) for categorical.

SSI = surgical site infection; VTE = venous thromboembolism; DVT = deep vein thrombosis; UTI = urinary tract infection; MI = myocardial infarction; CVA = cerebrovascular accident; LOS = length of stay; OR = operating room.

**Table S3**. Univariable Logistic Regression for Any 30-Day Complication

| **Characteristic** | **OR (95% CI)** | **p-value** |
| --- | --- | --- |
| Alloplastic (vs. autograft) | 1.05 (0.83 to 1.32) | 0.68 |
| Defect >5cm | 1.34 (1.15 to 1.56) | **<0.001** |
| Age (years) | 1.02 (1.01 to 1.02) | **<0.001** |
| Female sex | 0.89 (0.77 to 1.04) | 0.15 |
| Race |  | 0.074 |
| White | — |  |
| Black | 1.47 (1.13 to 1.90) |  |
| Asian | 1.02 (0.65 to 1.54) |  |
| Other | 0.78 (0.23 to 2.08) |  |
| Unknown | 1.01 (0.83 to 1.24) |  |
| Hispanic | 0.79 (0.48 to 1.23) | 0.31 |
| BMI (kg/m2) | 1.00 (1.00 to 1.01) | 0.628 |
| Obesity (BMI>=30) | 0.96 (0.82 to 1.13) | 0.643 |
| Diabetes | 1.56 (1.26 to 1.92) | **<0.001** |
| Current smoker | 0.86 (0.70 to 1.06) | 0.15 |
| Ventilator dependent | 12.8 (7.37 to 23.8) | **<0.001** |
| COPD | 1.75 (1.18 to 2.57) | **0.006** |
| CHF | 2.81 (1.37 to 5.65) | **0.006** |
| Hypertension | 1.55 (1.33 to 1.81) | **<0.001** |
| Dialysis | 2.87 (0.71 to 10.9) | 0.13 |
| Disseminated cancer | 1.26 (1.01 to 1.56) | **0.039** |
| Steroid use | 1.62 (1.28 to 2.04) | **<0.001** |
| Bleeding disorder | 2.57 (1.62 to 4.02) | **<0.001** |
| Preop transfusion | 6.72 (2.74 to 17.9) | **<0.001** |
| Functional dependence | 2.07 (1.53 to 2.79) | **<0.001** |
| ASA class |  | **<0.001** |
| I | — |  |
| II | 1.38 (0.66 to 3.38) |  |
| III | 2.54 (1.24 to 6.14) |  |
| IV | 5.77 (2.74 to 14.2) |  |
| Emergency case | 2.57 (1.87 to 3.53) | **<0.001** |
| Inpatient setting | 2.80 (1.53 to 5.76) | **<0.001** |
| Abbreviations: CI = Confidence Interval, OR = Odds Ratio | | |

OR = odds ratio; CI = confidence interval. Bold = p<0.05.

# **Table S4.** Secondary Outcomes: Adjusted ORs for Material Type, Defect Size, and Their Interaction. Each model adjusted for age, sex, obesity, diabetes, smoking, ventilator dependence, COPD, CHF, hypertension, dialysis, disseminated cancer, steroid use, bleeding disorder, preoperative transfusion, functional dependence, ASA class, operative time, emergency status, and inpatient setting.

| **Outcome** | **Predictor** | **Adjusted OR (95% CI)** | **p-value** |
| --- | --- | --- | --- |
| Any Complication | Alloplastic (vs. Autograft) | 1.43 (0.83-2.39) | 0.183 |
|  | Defect >5cm (vs. <=5cm) | 1.28 (1.04-1.57) | 0.018 |
|  | Alloplastic x Defect >5cm (interaction) | 0.59 (0.29-1.21) | 0.151 |
| Any SSI | Alloplastic (vs. Autograft) | 1.38 (0.33-4.00) | 0.599 |
|  | Defect >5cm (vs. <=5cm) | 1.56 (0.99-2.46) | 0.055 |
|  | Alloplastic x Defect >5cm (interaction) | 0.52 (0.10-2.89) | 0.422 |
| Surgical Complication | Alloplastic (vs. Autograft) | 1.08 (0.26-3.09) | 0.900 |
|  | Defect >5cm (vs. <=5cm) | 1.39 (0.91-2.13) | 0.124 |
|  | Alloplastic x Defect >5cm (interaction) | 0.63 (0.12-3.50) | 0.573 |
| Medical Complication | Alloplastic (vs. Autograft) | 1.35 (0.67-2.54) | 0.372 |
|  | Defect >5cm (vs. <=5cm) | 1.09 (0.83-1.42) | 0.526 |
|  | Alloplastic x Defect >5cm (interaction) | 0.71 (0.29-1.76) | 0.455 |
| Reoperation | Alloplastic (vs. Autograft) | 0.90 (0.38-1.89) | 0.800 |
|  | Defect >5cm (vs. <=5cm) | 0.99 (0.73-1.32) | 0.931 |
|  | Alloplastic x Defect >5cm (interaction) | 0.89 (0.30-2.74) | 0.842 |
| Readmission | Alloplastic (vs. Autograft) | 0.92 (0.40-1.86) | 0.836 |
|  | Defect >5cm (vs. <=5cm) | 1.04 (0.79-1.35) | 0.788 |
|  | Alloplastic x Defect >5cm (interaction) | 1.19 (0.46-3.23) | 0.724 |
| 30-Day Mortality | Alloplastic (vs. Autograft) | 2.00 (0.42-6.59) | 0.310 |
|  | Defect >5cm (vs. <=5cm) | 0.66 (0.30-1.35) | 0.262 |
|  | Alloplastic x Defect >5cm (interaction) | 0.90 (0.12-6.71) | 0.916 |
| Non-Home Discharge | Alloplastic (vs. Autograft) | 1.10 (0.61-1.90) | 0.749 |
|  | Defect >5cm (vs. <=5cm) | 1.13 (0.92-1.40) | 0.244 |
|  | Alloplastic x Defect >5cm (interaction) | 1.12 (0.54-2.36) | 0.765 |

Interaction term tests whether the effect of alloplastic material differs by defect size.

OR = odds ratio; CI = confidence interval.

**Table S5.** Alloplastic vs. Autograft: Subgroup Analysis by ASA Risk Category. Each model adjusted for defect size, age, sex, obesity, diabetes, smoking, ventilator dependence, COPD, CHF, hypertension, dialysis, disseminated cancer, steroid use, bleeding disorder, preoperative transfusion, functional dependence, operative time, emergency status, and inpatient setting.

| **Subgroup** | **Outcome** | **Alloplastic OR (95% CI)** | **p-value** |
| --- | --- | --- | --- |
| Low Risk (ASA I-II) | Any Complication | 1.64 (0.73-3.39) | 0.202 |
|  | Any SSI | 0.61 (0.03-3.25) | 0.644 |
|  | Surgical Complication | 0.57 (0.03-3.06) | 0.603 |
|  | Medical Complication | 2.59 (0.90-6.37) | 0.053 |
|  | Reoperation | 2.06 (0.66-5.30) | 0.163 |
|  | Readmission | 2.41 (1.04-5.10) | 0.029 |
|  | 30-Day Mortality | 0.00 (NA-Inf) | 0.997 |
|  | Non-Home Discharge | 1.35 (0.44-3.39) | 0.552 |
| High Risk (ASA III-IV) | Any Complication | 0.98 (0.65-1.45) | 0.926 |
|  | Any SSI | 0.99 (0.37-2.20) | 0.988 |
|  | Surgical Complication | 0.85 (0.32-1.88) | 0.722 |
|  | Medical Complication | 0.97 (0.58-1.57) | 0.912 |
|  | Reoperation | 0.68 (0.34-1.26) | 0.251 |
|  | Readmission | 0.76 (0.41-1.31) | 0.347 |
|  | 30-Day Mortality | 2.19 (0.73-5.58) | 0.125 |
|  | Non-Home Discharge | 1.23 (0.83-1.79) | 0.288 |

OR = odds ratio; CI = confidence interval.

**Table S6.** Alloplastic vs. Autograft: Stratified by Defect Size. Each model adjusted for age, sex, obesity, diabetes, smoking, ventilator dependence, COPD, CHF, hypertension, dialysis, disseminated cancer, steroid use, bleeding disorder, preoperative transfusion, functional dependence, ASA class, operative time, emergency status, and inpatient setting.

| **Defect Size** | **Outcome** | **Alloplastic OR (95% CI)** | **p-value** |
| --- | --- | --- | --- |
| Small (<=5cm) | Any Complication | 1.40 (0.81-2.35) | 0.220 |
|  | Any SSI | 1.40 (0.32-4.12) | 0.595 |
|  | Surgical Complication | 1.05 (0.24-3.08) | 0.937 |
|  | Medical Complication | 1.30 (0.64-2.46) | 0.445 |
|  | Reoperation | 0.92 (0.38-1.94) | 0.831 |
|  | Readmission | 0.93 (0.40-1.89) | 0.855 |
|  | 30-Day Mortality | 2.14 (0.44-7.35) | 0.276 |
|  | Non-Home Discharge | 1.12 (0.61-1.95) | 0.705 |
| Large (>5cm) | Any Complication | 0.85 (0.51-1.37) | 0.521 |
|  | Any SSI | 0.71 (0.21-1.87) | 0.532 |
|  | Surgical Complication | 0.68 (0.20-1.77) | 0.482 |
|  | Medical Complication | 0.90 (0.47-1.63) | 0.735 |
|  | Reoperation | 0.87 (0.38-1.79) | 0.732 |
|  | Readmission | 1.08 (0.57-1.92) | 0.802 |
|  | 30-Day Mortality | 2.43 (0.44-10.63) | 0.263 |
|  | Non-Home Discharge | 1.18 (0.73-1.88) | 0.481 |

OR = odds ratio; CI = confidence interval.

**Table S7.** Machine Learning Model Performance Comparison. 80/20 train/test split (Train N=2256, Test N=565). Outcome: any 30-day complication.

| **Model** | **AUC (95% CI)** |
| --- | --- |
| Logistic Regression | 0.737 (0.686–0.788) |
| LASSO | 0.733 (0.682–0.784) |
| XGBoost | 0.728 (0.676–0.781) |
| Random Forest | 0.698 (0.644–0.753) |

AUC = area under the receiver operating characteristic curve; CI = confidence interval.

LASSO = least absolute shrinkage and selection operator.
