# Supplementary material for: Material type versus defect size in adult cranioplasty: a multi-institutional analysis of 30-day outcomes and risk prediction modeling
Source: JPRAS Open. 2026 Aug 19;52:213–25. doi: 10.1016/j.jpra.2026.08.024 (PMC13571319; doi:10.1016/j.jpra.2026.08.024)
Supplement: Supplementary file 4 [file mmc1.docx]

**Supplementary Figure 1.** Forest Plot of Stratified Analysis by Defect Size (≤5 cm vs. >5 cm)
